# General Gorgas

**Published:** 1918-08

**Authors:** 


### General Gorgas.

Much conjecture is being made in the press as to what will become of Surgeon General Gorgas when he reaches the retiring age next October. Everywhere the medical press is uring that he be retained, retiring age or no retiring age—that the country needs his services now and that he is better prepared than ever to render acceptable service.

The Washington Post says that Surgeon General Gorgas is a miracle man. Hindenburg is known by the men he has sent forth to be slaughtered. Gorgas is known by the thousands whose lives he has saved. Once Havana was little better than a pest city. Yellow fever each year took from its citizens a frightful toll of death. When Cuba became "Cuba libre," Gorgas was put in charge of sanitation in Havana. He wrought wonders. He turned this pest hole into a health resort. His marvelous efficiency commanded the admiration of the world.

Came a time later when the United States decided to link two oceans. But the Panama Canal Zone, where thousands of Americans would have to labor at this stupendous task, was death's dinner table. It slew its thousands and its ten of thousands by means of the mosquito and the fly.

General Gorgas was sent there. And again General Gorgas wrought a wonderful thing. He came to grips with disease and pinned both shoulders of that gaunt wrestler to the mat. He cleaned up the Zone as he had cleaned up Havana. President Taft said that he "made possible the completion of the greatest industrial undertaking in the history of the world."

In South Africa thousands of miners in the Rand and Rhodesia were dying from pneumonia. The British government called on Gorgas to combat the disease. He responded. He conquered. He reduced the death rate to a minimum.

During the present war his efficiency has been demonstrated by the training within less than a year of a great army of doctors, nurses and hospital attendants to care for wounded and sick soldiers abroad and in this country.

So we cannot conceive of the retirement of General Gorgas. It would be a national calamity. Perhaps the President has not the technical right to reappoint him after his retiring age has been reached. Again we agree with the Medical Journal that Congress will pass proper legislation giving him that right.

This country needs General Gorgas more now than it has ever needed him. He is still active. He is at the height of efficiency because of his past experiences. Age has not affected his ability. In our opinion, it would be a grave mistake if red tape should strangle his services.

